# Quantitative evaluation of ^123^I-MIBG imaging in patients with myocarditis: impairment of cardiac neuronal function revisited

**DOI:** 10.1007/s12149-025-02120-w

**Published:** 2025-10-17

**Authors:** Lukas Kessler, Stephan Settelmeier, Kim M. Pabst, Tugce Telli, Zohreh Varasteh, Pedro Fragoso Costa, Walter Jentzen, Francesco Barbato, Hubertus Hautzel, Stephan Himmen, Christoph Rischpler, Tienush Rassaf, Ken Herrmann, David Kersting

**Affiliations:** 1 Department of Nuclear Medicine, University Hospital Essen, University of Duisburg-Essen, Hufelandstrasse 55, 45147 Essen, Germany; 2 Institute of Diagnostic, Interventional Radiology and Neuroradiology, University Hospital Essen, Hufelandstrasse 55, 45147 Essen, Germany; 3 Department of Cardiology and Vascular Medicine, West German Heart and Vascular Center, University Hospital Essen, Hufelandstrasse 55, 45147 Essen, Germany; 4 Department of Nuclear Medicine, Klinikum Stuttgart, Prießnitzweg 24, 70374 Stuttgart, Germany

**Keywords:** Myocarditis, Quantitative imaging, MIBG, Sympathic innervation

## Abstract

**Background:**

^123^I-MIBG has been shown to visualize impaired cardiac neuronal function, but data of this imaging modality in patients with acute myocarditis are scarce. Nonetheless, an association with reduced cardiac function has been observed previously. The aim of this study was to establish and evaluate semi-quantitative and quantitative parameters in ^123^I-MIBG scintigraphy and SPECT/CT in patients with acute myocarditis and identify associations with left ventricular ejection fraction (LVEF) and biomarkers.

**Methods:**

Eight patients with acute myocarditis and a gender and age-matched control group who underwent ^123^I-MIBG scintigraphy and SPECT/CT were retrospectively analysed. Semi-quantitative Heart-to-Mediastinum (*H*/*M*) ratio and washout rate were calculated, additionally SPECT/CT system calibration and a whole-heart-segmentation were used for absolute quantification of tracer uptake. ROC analysis for the prediction of acute myocarditis and correlation of imaging parameters with LVEF and serological biomarkers was performed.

**Results:**

Seven patients (87.5%) showed visually decreased tracer uptake. Planar imaging parameters showed significant differences compared to the control group (e.g. *H*/*M* ratio 1.6 ± 0.3 vs. 2.3 ± 0.8, *p* < 0.05), as well as multiple quantitative parameters e.g. SUVmean (1.7 ± 0.5 vs. 3.0 ± 1.0; *p* < 0.01). Additionally, correlation between imaging parameters and LVEF (e.g. SUVmax *r* = 0.85, *p* < 0.01) and NT-proBNP (e.g. *H*/*M* *r* = − 0.88, *p* < 0.05) was observed.

**Conclusion:**

^123^I-MIBG visualizes impairment of cardiac neuronal function in patients with acute myocarditis and is associated with reduced ejection fraction and elevated NT-proBNP. We could establish an absolute quantification approach that could offer novel diagnostic opportunities for disease assessment and risk stratification, which will be focused on further studies.

**Supplementary Information:**

The online version contains supplementary material available at 10.1007/s12149-025-02120-w.

## Introduction

Myocarditis is a medical condition characterized by inflammation of the myocardium of the heart. This inflammation can weaken the heart muscle, impairing cardiac function and leading to a range of symptoms and potential complications [1]. Due to heterogeneous clinical presentation, myocarditis remains a challenging diagnosis and up-to-date diagnostic approaches typically involve a combination of clinical evaluation, imaging, laboratory tests and invasive exclusion of coronary artery disease [2]. Non-invasive diagnostics are focused on ECG, echocardiography and cardiac magnetic resonance imaging (CMR), but various molecular imaging approaches have been investigated previously [3–5]. A rarely investigated imaging probe in this setting is metaiodobenzylguanidine (MIBG). MIBG is a compound that mimics norepinephrine and allows non-invasive visualization of cardiac sympathetic innervation and thereby cardiac neuronal dysfunction in heart diseases [6–9]. In recent years, ^123^I-MIBG scintigraphy has been shown to be a potential tool in the prediction of cardiac events and death in heart failure and thus gaining clinical relevance [6]. Although ^123^I-MIBG scintigraphy has only been scarcely investigated in patients with myocarditis. The only relevant study from 1998 showed an association of cardiac neuronal function with acute myocarditis in a small cohort [10]. The authors could show that decreased Heart-to-Mediastinum uptake ratios correlate with impaired left ventricular ejection fraction, thus reflecting cardiac neuronal dysfunction. Novel quantitative approaches in SPECT/CT imaging have shown promise in disease detection and identification of prognostic parameters for various cardiac diseases, but have not been evaluated in acute myocarditis so far [11–14]. Therefore, the utilization of semi-quantitative approaches in ^123^I-MIBG scintigraphy and SPECT/CT might enhance the diagnostic performance of myocarditis diagnosis and could play a valuable role in providing insights into the underlying pathophysiology and prognostic factors in myocarditis, ultimately aiding in the formulation of targeted treatment plans and improving patient outcomes.

Aim of this feasibility study was to evaluate established semi-quantitative measurements and novel SPECT/CT quantification for the assessment of cardiac sympathetic innervation in patients with acute myocarditis and correlate these imaging biomarkers with functional parameters and conventional biomarkers of cardiac function.

## Methods

### Patient population

Between June 2020 and January 2021, 8 patients (3 female/5 male; mean age 39 ± 17 years) received ^123^I-MIBG scintigraphy and SPECT/CT in the diagnostic work-up of myocarditis with a uniform imaging protocol at our institution. All patients underwent clinical examination, serum biomarker examination, ECG, ECHO and exclusion of coronary artery disease. Seven patients (87.5%) underwent CMR (Lake-Lousie Criteria). An age and gender matched control group was selected based on the following criteria: (a) Patients underwent ^123^I-MIBG planar scintigraphy and SPECT/CT of the thorax in early and late phase; (b) imaging due to primary staging with limited/curative disease extent in therapy-naïve patients or disease monitoring in patients with complete remission; (c) no cardiotoxic medication in the history; (d) no reported heart disease and preserved cardiac function. All patients gave written informed consent to the examination. Data were retrospectively analysed and anonymised prior to evaluation. The study was conducted in accordance with the Declaration of Helsinki and approved by the local institutional ethics committee (University of Duisburg-Essen, Ethics protocol number 25-12484-BO).

### Image acquisition and evaluation of ^123^I-MIBG images

A mean ± standard deviation (SD) of 321 ± 94 MBq of ^123^I-MIBG was intravenously administered. Planar whole-body scintigraphy and SPECT/CT images of the thorax were acquired 15 min and 4 h after tracer injection on a Symbia T2 or Symbia Intevo SPECT/CT system (both Siemens Healthineers. Erlangen, Germany). Semi-quantitative measurements, including Heart-to-Mediastinum (*H*/*M*) ratio and washout, were reported from planar images according to proposed guidelines [15]. For quantification, SPECT/CT systems were calibrated by phantom measurements based on previously described protocols for other radiotracers [11, 16]. A whole-heart segmentation method was used as previously described [11]; the calculated voxel-based activity concentration was decay-corrected and converted to standardized uptake values (SUVmean, max, peak) normalized to body weight. All images were acquired with a uniform camera and acquisition settings. In addition to conventional *H*/*M* ratio and tracer washout, quantitative SUVmean, tracer to background ratio (TBRmean), and SUV washout rate were calculated analog to planar established parameters:

$$\mathrm{SUVmean} H/M= \frac{\text{SUVmean heart}}{\text{SUVmean mediastinum}}$$

$$\text{SUV washout}=\left(\frac{\text{early SUVmean}-\text{late SUVmean}}{\text{early SUVmean}}\right)\times 100\%$$

Images were analyzed with Syngo.Via (Siemens Healthineers. Erlangen. German) and PMOD v4.2 (Zurich. Switzerland) for SPECT/CT quantification.

### Statistical analysis

Data were statistically analyzed with GraphPad Prism 10.2.3 for Windows (GraphPad Software, Boston, Massachusetts, USA). Continuous data are reported as mean ± SD. Correlation between imaging parameters and clinical parameters was evaluated by linear regression and the Spearman rho-method for nonparametric data. Kruskal–Wallis analysis of variance was used when comparing more than 2 groups as the omnibus test. Differences between nonparametric dates were calculated pairwise by Mann–Whitney-*U*-tests. Receiver–operater-curve analysis was performed for identification of cut-offs and optimal sensitivity (SE) and specificity (SP) for myocarditis by Youden index. *p* value < 0.05 were considered as statistically significant.

## Results

### Patient cohort

We included 5 male and 3 female patients with a mean age of 39 ± 17 years in the myocarditis cohort and 4 male/4 female in the control cohort with mean age of 45 ± 11 years (Table 1). Six patients exhibited reduced left ventricular ejection fraction (LVEF) with a mean of 45.6 ± 14.3 vs. 54.6 ± 5.5% in the control group (Table 1). Detailed baseline characteristics are shown in Table 1. Acute myocarditis was ultimately diagnosed in 5 patients by endomyocardial biopsy and in 3 patients by clinical and CMR imaging (Two positive main Lake-Louise criteria) results after acute ischemic heart disease was ruled out by coronary angiography. Summarized diagnostic criteria are shown in Supplemental Table 1.

**Table 1:** Baseline characteristics of myocarditis patients

	Mean ± SD or no. of patients (%)		*p* value
	Myocarditis	Control	
Age (y)	39 ± 17	45 ± 11	0.40
Gender (m/f)	5/3 (62.5/37.5%)	4/4 (50.0/50.0%)	> 0.99
BMI (kg/m^2^)	26.5 ± 5.1	25.5 ± 3.2	0.78
LVEF (%)	45.6 ± 14.3	54.6 ± 5.5	0.20
*Imaging/biopsy*			
CMR	7 (87.5)	–	–
Late gadolinium enhancement	6/7 (85.7)	–	–
EMB	5 (62.5)	–	–
*NYHA class*			
0	2 (25.0)	–	–
I	0 (0.0)	–	–
II	3 (37.5)	–	–
III	1 (12.5)	–	–
IV	2 (25.0)	–	–
*Biomarker*			
NT-proBNP (pg/ml)	445 ± 443 (range: 90–1443)	–	–
Creatine kinase peak (U/l)	202 ± 217	–	–
Troponin I peak (ng/l)	3113 ± 6414	–	–
LDH (U/l)	270 ± 67	–	–
Myoglobin (µg/l)	61 ± 51	–	–
*CVRF*			
Hypertension	1 (12.5)	–	–
Nicotine	2 (25.0)	–	–
Obesity	2 (25.0)	–	–
Prior CAD	2 (25.0)	–	–

### Comparison of imaging parameters

On visual interpretation, 7 patients with acute myocarditis (87.5%) showed decreased myocardial tracer uptake (Table 2). Furthermore, the established semi-quantitative parameters from planar imaging, *H*/*M* ratio and washout were significantly lower compared to control group (*H*/*M* 1.6 ± 0.3 vs. 2.3 ± 0.8, *p* < 0.05; washout 8.3 ± 19.2 vs. 32.5 ± 28.8; *p* < 0.05). Using absolute quantification only SUVmean (1.7 ± 0.5 vs. 3.0 ± 1.0; *p* < 0.01) and TBRmean (3.0 ± 0.8 vs. 5.0 ± 1.2; *p* < 0.01) showed significantly decreased values compared to control-group patients. Additionally, myocarditis patients had significantly lower quantitative SPECT washout rates compared to controls (17.2 ± 13.3 vs. 52.5 ± 18.6; *p* < 0.01) (Table 2).

**Table 2:** Comparison of Imaging parameters

	Myocarditis	Control	*p* value
*Planar imaging*			
Visually decreased uptake	7 (87.5)	0 (0.0)	< 0.01
*H*/*M*	1.56 ± 0.30	2.26 ± 0.79	< 0.05
Washout %^a^	13.7 ± 12.3	41.4 ± 7.4	< 0.01
*SPECT/CT*			
SUVmean	1.7 ± 0.5	3.0 ± 1.0	< 0.01
SUVpeak	3.8 ± 1.0	5.2 ± 1.9	0.28
SUVmax	4.8 ± 1.3	6.5 ± 2.3	0.18
TBRmean	3.0 ± 0.8	5.0 ± 1.2	< 0.01
SUV washout rate	17.2 ± 13.3	52.5 ± 18.6	< 0.01

^a^Decay corrected

### Correlation with functional parameters and biomarkers

Correlation of semi-quantitative and quantitative parameters with LVEF (Table 3), the main functional parameter, showed significant moderate to strong positive correlation of *H*/*M* (*r* = 0.74; *p* < 0.05), and all quantitative uptake values, e.g. SUVmax (*r* = 0.85; *p* < 0.01) and TBRmean (*r* = 0.81; *p* < 0.05). Planar washout rate and quantitative SUV washout rate showed both inverse, negative correlation to LVEF (Washout *r* = − 0.79; *p* < 0.05; SUV washout rate *r* = − 0.82; *p* < 0.05). Correlation with serological biomarker revealed only significant negative relationships with NTproBNP for *H*/*M* (*r* = − 0.88; *p* < 0.01), SUVmean (*r* = − 0.76; *p* < 0.05), TBRmean (*r* = − 0.86; *p* < 0.05) (Table 4). The other biomarkers did not show significant correlation.

**Table 3:** Correlation with LVEF

Parameter vs LVEF	Spearman *R* (95% CI)	*p* value
*Planar imaging*		
H/M	0.74 (0.11 to 0.95)	< 0.05
Washout %^a^	− 0.79 (− 0.96 to − 0.20)	< 0.05
*SPECT/CT*		
SUVmean	0.83 (0.29 to 0.97)	< 0.05
SUVpeak	0.75 (0.10 to 0.95)	< 0.05
SUVmax	0.85 (0.37 to 0.97)	< 0.01
TBRmean	0.81 (0.14 to 0.97)	< 0.05
SUV washout rate	− 0.82 (− 0.97 to − 0.28)	< 0.05

^a^Decay corrected

**Table 4:** Correlation with biomarker

Imaging vs biomarker	CK peak		Trop I peak		Myoglobin		LDH		NT-proBNP	
Spearman *R* and *P* value	*R*	*p*	*R*	*p*	*R*	*p*	*R*	*p*	*R*	*p*
*H*/*M*	0.52	0.2	0.612	0.12	0.57	0.2	− 0.095	0.84	− 0.88	< 0.01
Washout %^a^	− 0.04	0.9	− 0.14	0.75	− 0.18	0.71	0.024	0.98	0.43	0.30
SUVmean	0.43	0.3	0.69	0.07	0.64	0.14	− 0.024	0.98	− 0.76	< 0.05
TBRmean	0.53	0.19	0.67	0.08	0.64	0.14	− 0.07	0.88	− 0.86	< 0.05
SUV washout	− 0.08	0.85	− 0.43	0.30	− 0.04	0.94	0.24	0.58	0.48	0.24

^a^Decay corrected

### Diagnostic performance

ROC analysis of imaging parameters was performed for identification of cut-off values and respective sensitivity and specificity for the detection of myocarditis (Table 5). Quantitative parameters showed higher sensitivity and specificity compared with planar imaging parameters. Highest sensitivity and specificity were observed for SUVmean at a cut-off of 2.17 with SE 100.0% and SP 87.50% and SUV washout rate at a cut-off of 33.22 with SE 100.0% and SP 87.50%.

**Table 5:** ROC analysis of imaging parameters

	Cut-off	Sensitivity	Specificity	Likelihood	*p* value
*H*/*M*	1.65	87.5	62.5	2.3	0.02
Washout %^a^	21.48	80.0	75.00	3.2	0.03
SUVmean	2.17	100.0	87.50	8.00	0.006
TBRmean	3.66	85.71	87.50	6.9	0.004
SUV washout rate	33.22	100.0	87.50	8.0	0.004

^a^Decay corrected

### Case example

**Fig. 1:**
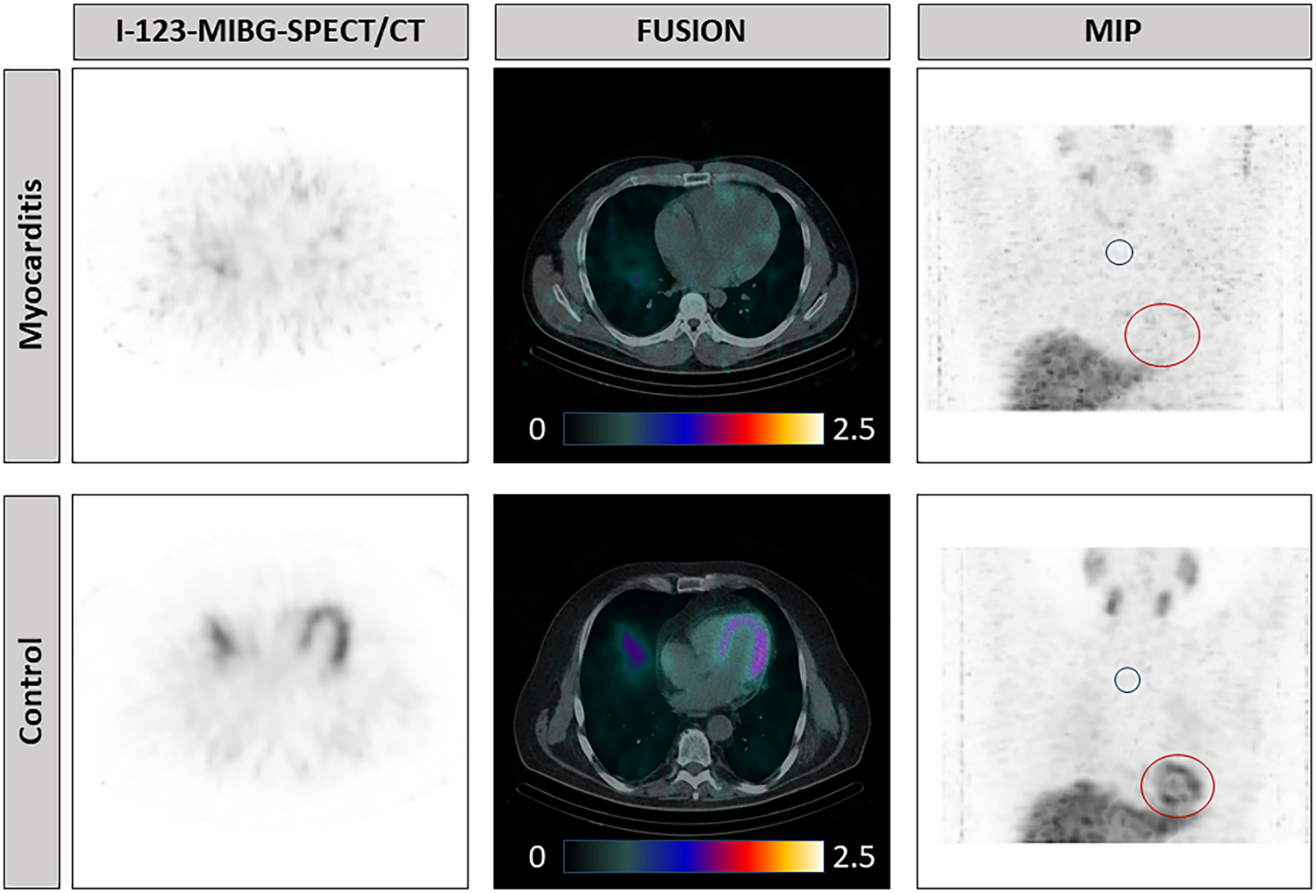
Case example of ^123^I-MIBG SPECT/CT in a patient with myocarditis and control patient 4 h p.i..MIP, maximum intensitiy projection. Color scale in KBq/ml

Figure 1 depicts a case of 42-year-old male patient, who presented with chest pain, heart failure and fever. Echocardiography showed reduced LVEF of 19% and diastolic dysfunction. Coronary artery disease was ruled out by coronary angiography. On ^123^I-MIBG SPECT/CT globally decreased tracer uptake was observed in the myocardium. Endomyocardial biopsy validated a lymphocytic myocarditis. A control patient with no known heart disease and physiological tracer distribution is shown for comparison (Fig. 1).

## Discussion

Up to date, a variety of imaging probes have been investigated in the diagnostic work-up of acute myocarditis with a focus on myocardial inflammation. Another approach in the last two decades has been the visualization of sympathetic myocardial innervation, especially ^123^I-MIBG imaging, which has been linked to patient outcomes. Our analysis aimed to evaluate quantitative ^123^I-MIBG SPECT/CT in patients with acute myocarditis as a potential diagnostic tool and the identification of relationships with left ventricular ejection fraction and serological biomarkers.

A study from 1998 was the first and only study to show decreased *H*/*M* ratios and thus impaired sympathetic innervation in patients with acute myocarditis on planar ^123^I-MIBG scintigraphy. In this cohort, we could reproduce this finding and further observed lower tracer washout rates in myocarditis patients, which has not been shown before. Additionally, novel quantitative parameters SUVmean, TBRmean and SUV washout rates aligned with these findings, but interestingly SUVmax and SUVpeak did not show significant differences compared to healthy controls. This could be technically explained by point-estimate measurement of hottest voxel/focal uptake values in affected myocardium with diffuse myocardial involvement, rather than average uptake of the heart measured by SUVmean or TBRmean. These data indicate that SUVmax and SUVpeak might not be sufficient metrics for this imaging methodology and mean-based measures (SUVmean/TBRmean) better capture global reductions in uptake and exhibit superior repeatability. Another explanation might be the limited power of this small cohort. Further, in accordance with current data on impaired sympathetic innervation, planar and quantitative imaging parameters correlate with decreased LVEF and elevated NT-proBNP levels as a surrogate marker for heart failure, but planar and quantitative washout rates did not show correlation with NT-proBNP. Myocardial ^123^I-MIBG washout is usually interpreted as a reflection of neuronal integrity and is usually increased in patients with impaired cardiac neuronal function due to heart failure [17]. In our cohort, we could see that myocarditis patients showed significantly lower washout rates (planar and quantitative) than the healthy control patients, which is contradictory compared to existing data in heart failure patients with reduced *H*/*M* and increased washout. Because washout is a fraction normalized to early counts, lower initial uptake attenuates the calculated washout rate, producing lower WR values independent of true clearance kinetics. Thus, the WR difference appears primarily driven by reduced early uptake rather than enhanced tracer retention and strongly influences the reliability of washout calculation in this setting [18]. A potential explanation for the lower washout rates could be that cardiac sympathetic nerves are damaged by myocardial inflammation leading to a reduction/loss of nerve density rather than downregulation of norepinephrine transporters [19, 20].

Other biomarkers than NT-proBNP did not show relationships with the investigated parameters. These findings primarily confirm impaired cardiac neuronal function in heart failure patients, which also applies to our cohort of patients with acute myocarditis.

In this small cohort, the diagnostic performance was not of primary interest, but we observed high diagnostic values for quantitative values comparable to established CMR protocols. Nonetheless, these results should be interpreted with caution due to the correlation of sympathetic innervation with heart failure, which biases this analysis.

Despite efforts for proper standardization of cardiac ^123^I-MIBG imaging, institutional variations of imaging protocols, camera systems and acquisition settings often do not allow extrapolation of calculated results from planar imaging [18, 21]. Additionally, correct ROI placement is difficult in individuals with very low ^123^I-MIBG tracer uptake, where the anatomical borders of LV cannot be identified with certainty, ultimately hampering the calculation of *H*/*M* and washout [18]. Therefore, absolute quantification from SPECT/CT offers multiple benefits compared to planar imaging. Our approach relies on a whole-heart segmentation, which enables better measurement of overall myocardial tracer uptake, but still relies on low resolution of non-contrast, low-dose CT scans, which limits regional segmentation. Further, this approach provides a more precise measurement of tracer uptake and could be applied to different centres and quantification results could be cross-referenced and compared between institutions.

Another key factor is that sympathetic innervation has already been linked to outcome variables in heart failure patients. Heart failure and dilated cardiomyopathy are known complications of myocarditis and, therefore, ^123^I-MIBG imaging could potentially play a role in initial risk stratification, which will be focused on in perspective studies. A potential integration in clinical workflows could be to perform baseline ^123^I-MIBG imaging in patients with suspected acute myocarditis after hemodynamic/clinical stabilization and stratify patients with higher denervation burden to closer rhythm surveillance. For longitudinal assessment, repeated ^123^I-MIBG-imagng at 3–6 months after baseline could document reinnervation or persistent denervation, and could guide treatment and monitoring alongside CMR, biomarkers, and ECG.

### Limitation

This study is obviously limited by the small cohort size. A major factor contributing to this is that ^123^I-MIBG is not a commonly used imaging modality in this setting, and tracer logistics often restrict timely imaging compared to widely available echocardiography and CMR. Nonetheless, during the COVID pandemic, alternative non-invasive diagnostic strategies were sought after, which led to few patients being examined in this period of time. Further, potential referral/selection bias could influence the data because patients who underwent ^123^I-MIBG may differ from the broader myocarditis population in severity, contraindications to CMR/FDG-PET or clinician preference, which could dilute or inflate observed associations and constrain generalizability. Additionally, the need for standardized imaging protocols and the establishment of quantitative SPECT/CT measurements contributed to the small cohort. Another limitation is the control group, which does not consist of healthy volunteers but of cancer patients who were classified as “heart healthy” based on file review and clinical documentation. We cannot exclude subclinical cancer- or therapy-related cardiac effects, especially in patients with adrenergic tumors, which could attenuate between-group differences or introduce residual confounding. To overcome these limitations prospective, multicenter studies are warranted to validate and improve the robustness of our findings.

## Conclusion

This is the first study to establish a quantitative SPECT/CT imaging approach with ^123^I-MIBG in patients with acute myocarditis. We validated previously postulated impairment of cardiac neuronal function, showing a correlation with heart failure quantities and providing novel metrices in this setting. Inflammation-mediated decrease of adrenergic function/nerve damage might be a key player in impairment of cardiac function consequently leading to long-term complications. Despite the shown feasibility, the diagnostic performance and potential prognostic value of quantitative ^123^I-MIBG in acute myocarditis need further investigation to overcome the mentioned limitations.

## Supplementary Information

Below is the link to the electronic supplementary material.

Supplementary file1 (DOCX 16 KB)

## Abbreviations

*H*/*M*: Heart/mediastinum ratio
LVEF: Left ventricular ejection fraction
ROC: Receiver operating characteristics
ROI: Region-of-interests
SPECT/CT: Single photon emission computed tomography/computed tomography
SUV(max, peak, mean): Standardized uptake values
TBR: Tracer-to-background ratio
^123^I-MIBG: ^123^I-meta-iodobenzylguanidine
CMR: Cardiac magnetic resonance imaging
ECG: Electrocardiography
