# Supplementary material for: Quantitative evaluation of 123I-MIBG imaging in patients with myocarditis: impairment of cardiac neuronal function revisited
Source: Ann Nucl Med. 2025 Oct 17;40(2):202–8. doi: 10.1007/s12149-025-02120-w (PMC12923399; doi:10.1007/s12149-025-02120-w)
Supplement: Supplementary file 1 — Supplementary file1 (DOCX 16 KB) [file 12149_2025_2120_MOESM1_ESM.docx]

**Supplement**

**Supplemental Tabl. 1. Additional diagnostic criteria per patient; + = positive criteria; - negative criteria; *CMR revised Lake-Louise Criteria 2018.**

| **Patient No.** | **Cardiac Biomarkers** | **Fever** | **CAD (prior)** | **Echo** | **CMR*** | **EMB** |
| --- | --- | --- | --- | --- | --- | --- |
| **1** | **+** | **+** | **-** | **(-/+)** | **T2+/T1+** | **N.A.** |
| **2** | **+** | **+** | **-** | **-** | **T2+/T1+** | **+ (lymphocytic)** |
| **3** | **+** | **+** | **(+)** | **+** | **T2+/T1+** | **+ (lymphocytic)** |
| **4** | **+** | **-** | **-** | **+** | **T2-/T1+** | **+**  **(toxic)** |
| **5** | **+** | **+** | **-** | **+** | **-** | **+ (lymphocytic)** |
| **6** | **+** | **-** | **(+)** | **+** | **T2+/T1+** | **N.A.** |
| **7** | **+** | **+** | **-** | **-** | **T2+/T1+** | **N.A.** |
| **8** | **+** | **-** | **-** | **+** | **T2+/T1+** | **+ (lymphocytic)** |
